# The Spatial Dynamics of Predators and the Benefits and Costs of Sharing Information

**DOI:** 10.1371/journal.pcbi.1005147

**Published:** 2016-10-20

**Authors:** Matthieu Barbier, James R. Watson

**Affiliations:** 1 Centre for Biodiversity Theory and Modelling, National Centre for Scientific Research(CNRS), France; 2 Stockholm Resilience Centre, Stockholm University, Sweden; 3 College of Earth, Ocean and Atmospheric Sciences, Oregon State University, USA; CNRS, FRANCE

## Abstract

Predators of all kinds, be they lions hunting in the Serengeti or fishermen searching for their catch, display various collective strategies. A common strategy is to share information about the location of prey. However, depending on the spatial characteristics and mobility of predators and prey, information sharing can either improve or hinder individual success. Here, our goal is to investigate the interacting effects of space and information sharing on predation efficiency, represented by the expected rate at which prey are found and consumed. We derive a feeding functional response that accounts for both spatio-temporal heterogeneity and communication, and validate this mathematical analysis with a computational agent-based model. This agent-based model has an explicit yet minimal representation of space, as well as information sharing about the location of prey. The analytical model simplifies predator behavior into a few discrete states and one essential trade-off, between the individual benefit of acquiring information and the cost of creating spatial and temporal correlation between predators. Despite the absence of an explicit spatial dimension in these equations, they quantitatively predict the predator consumption rates measured in the agent-based simulations across the explored parameter space. Together, the mathematical analysis and agent-based simulations identify the conditions for when there is a benefit to sharing information, and also when there is a cost.

## Author Summary

When should we work together and when should we work alone? This question is central to our efforts to understand social and ecological systems alike, from lions hunting in the Serengeti to fishermen searching for their catch. Here, we develop a mathematical modeling framework to identify the essential spatial factors controlling the benefits and costs of sharing information. Our approach marries computation with mathematical analysis, and our results highlight that it is only under certain spatial conditions that information sharing is a useful cooperative strategy. Notably, we find conditions for which fully collective and fully individual search are both attractive.

## Introduction

Predators in numerous systems share information with one another to optimize their individual and collective gains [1]. These information-sharing cliques tend to form predatory packs or groups [2]. So too in social-ecological systems such as fisheries, where fishermen are sometimes known to share information with one another about the location of target species aggregations, and sometimes not [3, 4]. Critically, the benefits and costs of sharing information depend on the spatial characteristics of the system. For example, fishermen that share information tend to target highly ephemeral and migratory species like salmon [5]. In contrast, sessile and slow moving species tend to be harvested by secretive and even territorial fishermen [6]. Further, information sharing need not be voluntary, and there are numerous examples of predators copying the location of others [e.g. 5]. Hence, quantifying the relationship between the benefits and costs of information sharing and the spatial characteristics of the environments in which predators search for and capture prey, is important if we are to deepen our understanding of group formation and cooperation in social and natural systems alike.

An important consequence of space and information sharing is the potential inaccuracy of ecosystem models used to design management policies [4, 7, 8]. For instance, many population and ecosystem models used to inform policy on land and in the sea currently ignore or have overly simple parametric representations of predator and prey social and spatial behavior. In these models, it is commonly assumed that per-capita predator consumption depends only on prey abundance. This is reflected in the mathematical functions used to describe predator consumption: Type I (linear), Type II (saturating) and Type III (sigmoidal) functional responses [9, 10, 11]. This is due to the ecological legacy of these functions, having been well explored empirically and mathematically [12, 13, 14]. In some limiting cases, these simple functions can accurately represent aggregate feeding rates seen in nature [15]. However, all these feeding functions assume that the rate at which predators *encounter* (if not always *consume*) prey is linearly proportional to the density of the prey [2, 16]. In more complex spatial settings and when information sharing occurs, encounter rates are non-linear and as a consequence, these models will produce inaccurate individual and group feeding rates. This is acknowledged by ecosystem modelers themselves [17], and yet we remain limited in our ability to model predator group behavior in such contexts.

Agent-based models of predators searching for prey [e.g. 13, 18, 19] have been used to describe encounter rates, as they emerge from more realistic predator movement rules. Indeed, multiple models exist for predator search patterns, such as random [e.g. 20, 21], Lévy [e.g. 22] and correlated random walks [e.g. 23] to name a few. These studies deepen our understanding of the role space plays in the search process, but are limited in terms of accounting for both consumption of prey once found, and the impact of information sharing on the spatial distribution of predators on a given landscape. Together, both factors define the balance between the benefits and costs of information sharing: information sharing can reduce the time it takes to find prey, but it also comes at a cost, as prey is shared too.

Here, our goal is to assess the interacting effects of spatio-temporal heterogeneity and information sharing on predator consumption rates. In order to do so, we have developed a general mathematical model of predator foraging, accounting for space implicitly using a few key parameters. These parameters are timescales for search, consumption and prey mobility, which can be measured or computed independently for a given spatial setting, and from which we derive the benefits and costs of exploration, exploitation and information sharing. We note that these results do not address the strategic choices of predators or the evolution of cooperation [24]. Instead, we provide an extended form of the feeding functional response, incorporating the level of information sharing and spatial characteristics of both prey and predator. This allows us to compute the payoff, in terms of foraging efficiency, of a given information sharing behavior at the group level and for the individual. These payoffs are the foundation on which future evolutionary analyses could be performed, building off works on cooperation that use minimal representations of space [e.g. 25, 26, 27].

To complement the mathematical theory, we also developed a spatially explicit agent-based model (ABM) of predators and their prey, which we used to validate the analytical model and derive its abstract parameters from more intuitive individual processes. Even though the dynamics in the analytical model are encoded in a few behavioral states and a single spatial correlation metric, they quantitatively predict predator consumption rates measured in the ABM across the entire parameter space that we explored. Furthermore, and critically, very different simulation settings result in equivalent predation efficiencies, as long as they are characterized by the same key timescales. This means that the (spatial) assumptions of the ABM have limited impact, and that the mathematical analysis is far more general, being valid for a large range of predator-prey / consumer-resource systems, as modeled by other (perhaps more complex) ABMs or measured with empirical data.

## Behavioral Modeling of Predators

### Single-predator Feeding Function From Behavioral States

The first step to developing a mathematical feeding function that accounts for both space and information sharing is to view predator and prey interactions in terms of the rates at which predator behavioral states change. For example, let *s* be the fraction of a predator population that is moving in search of its prey, which is organized in distinct patches, and *h* be the fraction of predators currently “harvesting” or consuming prey. In the simple case of independent predators, there are no other states and *s* = 1 − *h*. Equivalently, since the population is for now assumed to be homogeneous, *s* and *h* can be seen as the fraction of time spent searching and consuming by a single predator.

**Fig 1:**
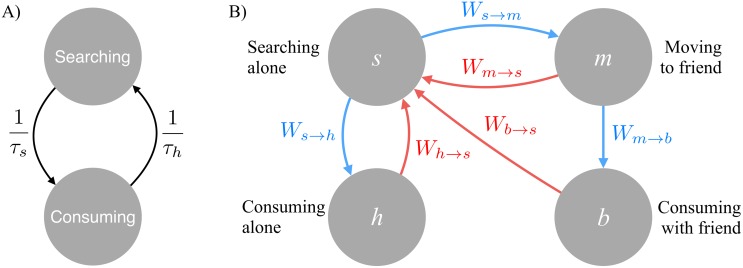
Schematics for A) a simple behavioral-state model of predators and their interaction with their prey; and B) for the predator behavioral state-change model, accounting for information sharing.

Furthermore, let us define *τ*_*s*_ the expected time taken for an individual predator to find a prey-patch (otherwise known as the first-passage time), and *τ*_*h*_ the patch handling time or expected time to consume all prey in a patch. The rate at which a searching predator finds a prey patch is then 1/*τ*_*s*_, and the rate at which a feeding predator returns to searching is 1/*τ*_*h*_. This simple behavioral state change model is depicted in Fig 1A. At steady state, the fluxes between behavioral states are equal:

$$\begin{array}{c} s\frac{1}{\tau_{s}}=h\frac{1}{\tau_{h}}. \end{array}$$ (1)

Furthermore, the consumption rate *H* (in units of prey per time) averaged over a foraging period will be proportional to *h*, the fraction of time spent consuming rather than searching. Let us denote by H* the maximal value attained when *s* = 0, a case where prey is abundant everywhere and can be found instantly. Using *s* + *h* = 1, we can write the functional response as

$$\begin{array}{c} \frac{H}{H^{*}}=\frac{h}{h+s}=\frac{1}{1+\frac{s}{h}}=\frac{1}{1+\frac{\tau_{s}}{\tau_{h}}}. \end{array}$$ (2)

Clearly, the feeding rate of a predator is inversely proportional to *τ*_*s*_/*τ*_*h*_, how long it takes to find prey patches relative to how long it takes to consume them. If we further assume that the rate at which predators encounter prey patches (1/*τ*_*s*_) is proportional to the density of prey, and there is no influence of predator group behavior, the expression on the far right takes the form of a classical Type II functional response. Alternatively, if the encounter rate is convex in prey density, rather than linear, then a Type III function is found instead.

### Behavioral Dynamics with Information Sharing

In order to explore information sharing amongst predators, we modify the simple behavioral state model introduced above (Fig 1A). First, let us redefine the two behavioral states *s* and *h* above as searching *alone* and consuming a patch *found by oneself*. Likewise, *τ*_*s*_ and *τ*_*h*_ become the typical search time and consumption time for a predator alone, not accounting for information sharing and group dynamics yet. Furthermore, we add *τ*_*l*_ the typical time during which a prey patch can be exploited before it moves. This introduces an element of landscape stochasticity or change, representing the dynamic nature of prey distributions found in a number ecological systems on land, in lakes and in the sea [28].

These three quantities—*τ*_*s*_, *τ*_*h*_ and *τ*_*l*_—play a key role in our analysis, as they can be independently measured or computed from the spatial characteristics of the individual predator and prey. Thus, they interface between the generic model presented here and any specific description of how predators and prey move and interact. In the next section, we develop an agent-based model to demonstrate how these key timescales can be computed from measurable quantities, albeit in a simplified abstraction of reality. We further expand on these key timescales in the discussion, describing their real world analogues for a range of natural and human systems.

In addition, there are two more parameters that control the benefits and costs of information sharing: *N* the number of predators foraging in the same area, and *λ* their propensity for sharing information, which we assume here to be identical for all predators. The consequences of relaxing this last constraint is explored in the Supplementary Online Information (SOI) section “Agent Level Mathematics”. The process of searching for prey patches remains solitary, but predators can now broadcast the location of a prey patch when they find one. This reflects group predator behavior found in social insects such as honey bees [29] as well as scavenger mammals such as hyenas [30]. We must then consider two new behavioral states: *m* which is the fraction of predators moving toward a patch whose location has been broadcast, and *b* the fraction of “bound” predators having reached the patch and are consuming prey with their caller. Clearly, the foraging efficiency is now the total fraction of time spent consuming prey whether one has found a patch or been called to it:

$$\frac{H}{H*}=h+b$$ (3)

Finally, we define the rates of change between these behavioral states: the rate at which lone searchers find and start consuming prey *W*_*s* → *h*_, the rate at which lone searchers get a call from and start moving towards another predator *W*_*s* → *m*_, the rate at which predators reach their caller *W*_*m* → *b*_, and the rate at which all these predators revert back to searching alone: *W*_*h* → *s*_,*W*_*m* → *s*_,*W*_*b* → *s*_. This new behavioral state model is depicted in Fig 1B and its associated parameters are listed in Table 1. What remains is to derive formulae for the different rates of behavioral state change. Starting with the rate at which lone searchers encounter a new prey patch:

$$W_{s\to h}=\frac{1}{\tau_{s}}$$ (4)

**Table 1:** Table of the parameters and variables of the general mathematical model.

Parameters:	
*N*	Number of predators
*λ*	Communication propensity
H*	Consumption/harvest rate for lone predator on prey patch
Timescales:	
*τ*_*s*_	Search time (lone predator)
*τ*_*l*_	Landscape (prey) mobility time
*τ*_*h*_	Handling time (lone predator)
Behavioral states:	
*s*	Lone searcher
*h*	Consuming (or harvesting) a patch that one found
*m*	Moving toward the source of a call
*b*	Consuming a patch found by one’s caller
Derived quantities:	
*W*_*i*→*j*_	Flux from behavioral state *i* to state *j*
*W*_*L*_	Flux back to the lone searcher state
*n*_*p*_	Expected number of predators consuming the same patch

Next is the rate at which lone searchers receive a call and start to move toward a patch:

$$W_{s\to m}=N\lambda sW_{s\to h}.$$ (5)

This rate is proportional to *N*×*s* the number of searchers, and to *W*_*s* → *h*_ the rate at which one of these searchers will discover a patch and switch from state *s* to *h*. As mentioned above, the factor *λ* quantifies the strength of social interactions, here conceptualized as the probability that a predator broadcasts information upon discovery of a prey patch.

Next, the rate at which predators reach their caller is the inverse of the expected travel time between two predators *τ*_*d*_:

$$W_{m\to b}=\frac{1}{\tau_{d}},$$ (6)

where, as a first approximation, *τ*_*d*_ can be taken as a constant, for example fixed to unity (meaning that other timescales are measured in units of *τ*_*d*_). In the SOI section “Distance between Predators”, we discuss a more elaborate derivation of *τ*_*d*_ which is necessary only for precise quantitative agreement with the ABM simulations, or with empirical data.

Finally, we define *W*_*L*_, the rate at which a predator is interrupted while exploiting a patch, either because the latter is depleted or has moved. This rate *W*_*L*_ accounts for the reversion from all other states to the lone searching state:

$$W_{h\to s}=W_{m\to s}=W_{b\to s}=W_{L}.$$ (7)

To compute it, we need two timescales. First, *τ*_*l*_ the expected time between a patch being discovered and it moving away. Second, *τ*_*h*_/*n*_*p*_ the expected time it takes to deplete the patch given a number *n*_*p*_ of predators consuming together (we assume here a simple inverse proportionality, but this is easily adapted to represent interference or cooperation between predators on a patch, without qualitatively affecting our results). These two timescales are combined in a Poisson process approximation: assuming that the probability of the patch *not* moving in the time interval [0,*t*] is exp(−*t*/*τ*_*l*_), and its probability of *not* being depleted yet is exp(−*tn*_*p*_/*τ*_*h*_), then the probability of both conditions being verified is the product exp(−*t*/*τ*_*l*_−*tn*_*p*_/*τ*_*h*_), but it is also exp(−*W*_*L*_ *t*) by definition. Hence, we can write:

$$W_{L}=\frac{1}{\tau_{l}}+\frac{n_{p}}{\tau_{h}}.$$ (8)

In the previous paragraph, we introduced an important new quantity: *n*_*p*_, the number of predators consuming at the same patch simultaneously. Deriving *n*_*p*_ is in fact the most intricate part of this analysis, as this quantity encodes the main relevant spatial and temporal correlations in the system, and thus represents non-mean-field dynamics in this otherwise space-less model. One derivation is given in the SOI section “Refined Occupancy Approximation”, but a basic intuition can be obtained from the following approximations, which hold only in simple limits:

$$n_{p}\approx \{\begin{array}{cc} N & \lambda \to 1,\\ \left(1+\frac{b}{h}\right) & \lambda \ll 1. \end{array}$$ (9)

If *λ* = 1, we expect all the predators to be fully correlated in space and time, and form a pack that always consumes the same prey. In such a situation *n*_*p*_ ≈ *N*. As *λ* becomes smaller however, time correlations become negligible and it is possible to use the time-averaged values *b* and *h* in *n*_*p*_ ≈ 1 + *b*/*h*, where *b*/*h* gives an estimate of the number of predators consuming at the same patch in addition to its finder (assuming a patch is not independently found by multiple searchers).

Given these formulae for the rates of change of the predator behavioral state occupation probabilities, it is possible to describe the evolution of these states in the predator population over time. In the long time limit, it converges toward a stationary point, where all the out- and in-fluxes between states are balanced:

$$s\left(W_{s\to h}+W_{s\to m}\right)=h\;W_{h\to s}+m\;W_{m\to s}+b\;W_{b\to s}$$ (10)

$$\begin{array}{cc} hW_{h\to s} & =s\;W_{s\to h} \end{array}$$ (11)

$$\begin{array}{cc} m\left(W_{m\to s}+W_{m\to b}\right) & =s\;W_{s\to m} \end{array}$$ (12)

$$\begin{array}{cc} b\;W_{b\to s} & =m\;W_{m\to b} \end{array}$$ (13)

This system can be reduced to a single equation over *s*, the fraction of predators searching alone:

$$\frac{1}{\tau_{s}}\left(N\lambda s^{2}+s\right)=\left(1-s\right)W_{L},$$ (14)

where *W*_*L*_ is a function *W*_*L*_(*τ*_*l*_,*τ*_*h*_,*s*) and all other terms are constant. Finally, the consumption rate for a predator in the group can itself be expressed using only *s*:

$$\frac{H\left(\tau_{s},\tau_{h},\tau_{l},N,\lambda \right)}{H*}=h+b=\left(\frac{1}{\tau_{s}}+\lambda \frac{Ns}{1+\tau_{d}W_{L}\left(\tau_{l},\tau_{h},s\right)}\right)\frac{1}{W_{L}\left(\tau_{l},\tau_{h},s\right)}s$$ (15)

The term 1/*τ*_*s*_ describes the baseline (single predator) success rate: it decreases with *τ*_*s*_, which can be thought of as the “cost” or difficulty of exploration, and increases with prey abundance (although it is a non-monotonic function of prey patchiness at a constant level of coverage, as detailed in the SOI section “Population Level Mathematics”). To this individual baseline, the second term within the large parenthesis describes the benefit of information sharing, and vanishes when *λ* = 0. The whole expression is modulated by 1/*W*_*L*_, the expected time during which a patch is available for consumption, and thus the effective “value” of each patch. This term increases with *τ*_*l*_ and *τ*_*h*_, that is for less mobile or richer patches. It is also a strictly decreasing function of *λ* the amount of information sharing, as a result of faster depletion, which is part of the *cost* of sharing. However, for small *τ*_*l*_ (very mobile prey) this variation is negligible, since mobility overtakes depletion as the main cause for returning to the searching-alone state. In other words, prey mobility discounts the cost of information sharing.

In conclusion, solving Eq (14) allows to us compute *H* in Eq (15), the consumption rate of any predator in the group given its environment, encoded by the key timescales *τ*_*s*_, *τ*_*h*_ and *τ*_*l*_, the number of predators *N* and their propensity toward information sharing *λ*. It is easily solvable numerically, and thus is a fast alternative to agent-based simulations. However, due to the complexity of the full expression of *n*_*p*_, the expected number of predators consuming prey at the same patch, it does not have an explicit solution. In our SOI section “Solvable Limits”, we discuss certain limiting cases where simplifying assumptions can be made to obtain explicit results. While these can contribute some insight into parts of the model, we show that none of these partial solutions can reflect the full phenomenology of this model or our ABM simulations below. Finally, in SOI section “Agent Level Mathematics” we show that this model is naturally extended to compute individual consumption rates in the case of heterogeneous agents, especially when they differ by their communication strategy *λ*.

## Agent-Based Model

**Fig 2:**
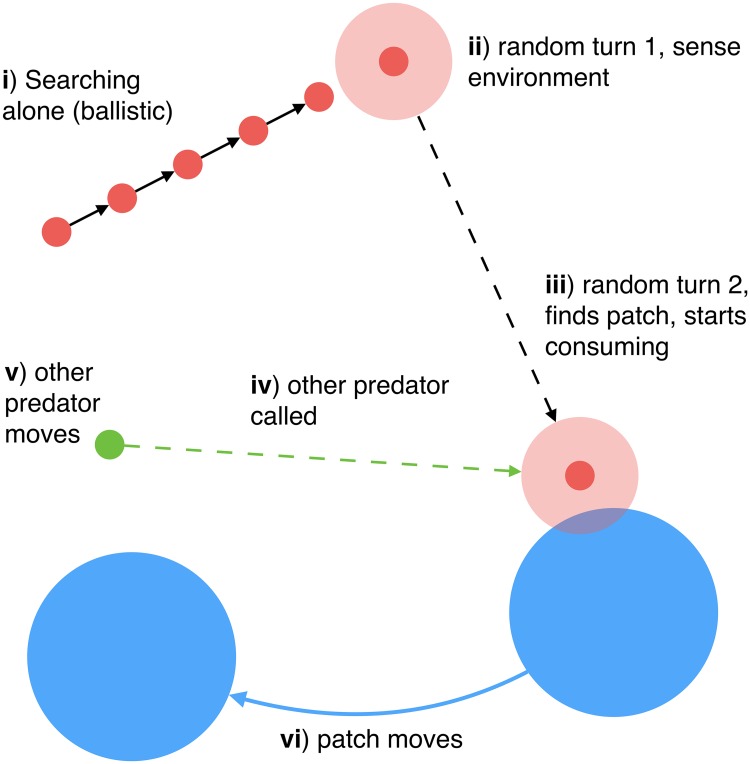
A cartoon describing our agent-based model of predators and prey. There are six main steps: (I) predators rapidly traverse the domain by moving in a straight line; (II) they randomly stop to sense their local environment, and pick a new direction if no prey patch is found within their sensory radius; (III) if a prey patch is found, the predator moves towards its centre and consumes prey from it; (IV) if a predator has a social tie with another predator who is at a prey patch, then information may be passed between the two, (V) informed predators move towards the nearest prey patch whose location they know; (VI) prey patches make random jumps at a given rate.

**Fig 3:**
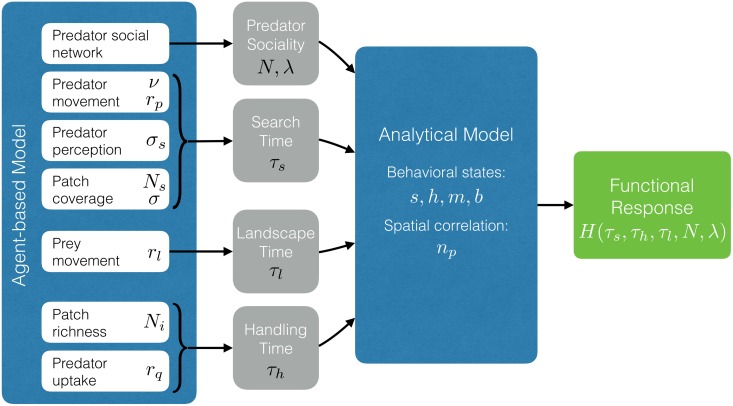
General schema of the relationship between the parameters in the numerical agent-based model and in the mathematical behavioral model. Parameters of the agent-based model represent those that could be measured empirically. These are then used to estimate the key parameters of the mathematical model (grey circles) following the formulae presented in the main text. The analytical model then uses these parameters to calculate the rates of change of predator behavioral states and the spatial correlation metric, all of which are required to derive a functional response that describes the consumption rate as a function of information sharing, and the spatial characteristics of the environment.

In addition to the general behavioral state model described above, we developed a spatially explicit, computational agent-based model (ABM) of predators and their prey. For a better qualitative understanding of how the ABM works, we refer the reader to the schematic in Fig 2, to our Supplementary Online Information where several movies show different simulation experiments (SOI: Movies), and to Table 2 where the parameters of the ABM are listed. This ABM allowed us to relate the abstract timescales of the mathematical analysis above (Table 1) to more concrete processes and properties that might be observed and measured in real-world systems (see Fig 3).

**Table 2:** Table of the main agent-based model parameters. In addition, one must specify the social network that governs communicative behavior among the agents (e.g. fully connected with homogeneous edge weight *λ*, or divided into multiple groups with edge weight 1 within a group and 0 between groups).

Domain:	
*X*	spatial scale of the domain
Prey:	
*N*_*s*_	number of patches
*N*_*i*_	number of prey items per patch
*σ*	radius of circular patch
*r*_*l*_	landscape change rate (per unit time probability of patch movement)
Predators:	
*N*	total number of predators
*σ*_*s*_	sensory radius
*v*	predator speed
*r*_*q*_	rate at which prey in patches are consumed
*r*_*p*_	turning rate (per unit time probability of making a random turn)

The ABM operates on a 2D landscape with periodic boundaries. On this landscape are a given number *N*_*s*_ of circular prey patches, a fish school for example, with a defined radius *σ*. Within these patches are a number of uniformly distributed prey items, *N*_*i*_, that could for instance represent individual fish within a school. The radius and number of prey items per patch is the same across patches. Prey mobility is important for our purposes only inasmuch as it limits the time that a predator can spend exploiting the same patch; we could let patches move so that predators could randomly lose their track, but for simplicity we simply let patches disappear with probability per unit time *r*_*l*_. However, we wish to conserve the total fraction of the landscape occupied by patches, and therefore we introduce a new patch at a random location every time one vanishes. Thus, the landscape change timescale is simply:

$$\tau_{l}=\frac{1}{r_{l}}.$$ (16)

A number *N* of predators is also found on this landscape, searching for prey patches. When a patch is found, they consume prey items within at rate *r*_*q*_. The expected patch handling timescale is:

$$\tau_{h}=\frac{N_{i}}{r_{q}},$$ (17)

We note that *r*_*q*_ = H* the maximal consumption rate discussed since Eq (2), since the average consumption rate over a simulation run is clearly at most *r*_*q*_. For increased realism, *r*_*q*_ could vary during consumption: for instance, a patch might be quickly depleted at first, then become harder to exploit. In that case, it could be advantageous for predators to leave a patch after a time *τ*_*h*_ that does not correspond to full depletion (as above) but instead optimizes their consumption rate. As this complicates both the simulations and the analysis, we leave this possibility to further inquiry.

In many systems, predators search for prey by alternating between fast travel between prey habitats, and intensive search (slow movement) at these habitats [31]. This is exhibited throughout nature, where numerous predator species switch between strict transiting/migration and feeding behaviors, and in social-ecological systems too, for example fishermen searching for ephemeral target-catch, such mobile pelagic species such as anchovy [32]. Multiple models exist to capture these predator search patterns, such as random [e.g. 20, 21], Lévy [e.g. 22] and correlated random walks [e.g. 23]. The qualitative feature shared by all these various models is an alternation between long-range movement and focused search within a local area. The simplest and most tractable model that retains this property is intermittent search [19]: predators move ballistically—in a straight line—at a constant speed *v*, with random direction changes made with probability per unit time (turning rate) *r*_*p*_. In-between two ballistic segments representing long-range movement, a predator spends one time-step exploring its surroundings to find prey, as represented by a sensing radius *σ*_*s*_. If there is no prey patch within that radius, it moves ballistically in the new direction. These three parameters, together with prey patch number *N*_*s*_ and radius *σ*, determine the search (first-passage) time *τ*_*s*_. To compute it, we build off work developed by Benichou et al. 2011 [19], who mathematically analyzed this type of search for only one prey patch and one predator. We provide a simple extension to account for multiple prey patches, which is presented in the SOI section “Intermittent Search”.

Predators can share information with one another about the location of prey patches: if two predators have a social tie, and one has found a prey patch but the other has not, information is shared between the predators with probability equal to *λ* the strength of the tie. In this situation, the predator receiving information moves towards the other predator. The social network is recorded in the form of a symmetric matrix of tie strengths. For simplicity, we mainly discuss two network structures: a fully connected graph where all ties have strength *λ* ∈ [0, 1], or a binary matrix where 1’s identify a social tie and 0’s the absence of a social tie. However, the model extends to arbitrary networks. Here, the predators’ communication range is assumed to encompass the whole landscape, and *N* is therefore the number of predators *within communication distance of each other*, whether randomly, due to coordination, or to extraneous social factors such as pack size.

If the behavioral state model described in the previous section is correct, the output of the ABM—namely the expected catch rate of a predator—should depend on its parameters only through the timescales which we derived, as well as *λ* and *N*. This we indeed show in SOI section “Validation of Analytical Results”, meaning that the parameter space has a much lower effective dimension than it first appears to have. While *τ*_*l*_ and *τ*_*h*_ have simple expressions in terms of the ABM parameters, *τ*_*s*_ is more complicated, reflecting the fact that our implementation puts an emphasis on details of the search process. Among the parameters influencing *τ*_*s*_, the most obvious one is the sensing radius *σ*_*s*_, which has an unambiguous influence on search difficulty, and affects nothing else. Thus, we generally explore the parameter space of the ABM by using *σ*_*s*_ as a control variable, fixing the total prey coverage *N*_*s*_ *πσ*^2^ and predator velocity *v*. Furthermore, in all simulations we use the turning rate *r*_*p*_ that minimizes *τ*_*s*_ for a single predator. We acknowledge that in nature, there is no guarantee that predators will have such optimal spatial behavior, and that the turning rate *r*_*p*_ will likely evolve together with the social structure of the predator population (encoded here by *N* and *λ*). However, we do not explore here the consequences of changing *r*_*p*_, and simply use the turning rate that minimizes *τ*_*s*_ as a way to be consistent across simulation experiments, and to speed them up.

## Simulation Experiments

We implemented the computational ABM using the Julia language (www.julialang.org), running several simulation experiments. Initially, these were used to vet the mathematical model, as presented in the SOI section “Validation of Analytical Results” and ultimately we found strong qualitative and quantitative agreement between the results of the ABM and the mathematical model. This success was especially important for subsequent simulation experiments, as it showed that the parameter space of the ABM could be efficiently explored along only three dimensions: those present in the mathematical model, i.e. first-passage time *τ*_*s*_, patch handling time *τ*_*h*_ and landscape change timescale *τ*_*l*_.

First, we performed a number of two-predator simulations, with the objective of measuring how the dimensionless ratio *H*/H* varies with predator information sharing. H* can be thought of as the maximal consumption rate obtained if prey-patch encounters are instantaneous and as a consequence predators consume prey constantly. Hence *H*/H* provides an estimate of the “efficiency” of the predators given their level of information sharing. We also mapped out the full functional response of the predators by measuring their consumption rates when systematically varying the landscape change timescale *τ*_*l*_, through changes in the rate at which prey patches move *r*_*l*_; the patch handling time *τ*_*h*_, through changes in the rate at which predators consume prey *r*_*q*_; and the first-passage time *τ*_*s*_ by varying *σ*_*s*_, the predator sensing radius.

This numerical implementation the ABM was also used to explore situations that involved more predators. However, these simulations were extremely computational demanding. Hence, in order to proceed and investigate situations with large numbers of predators, we used the mathematical model instead. We were then able to compute consumption rates for arbitrarily large numbers of predators. This allowed us to study the role of group size in maximizing individual consumption rate. This can be done in a number of ways, for example by varying *N* to find, as a function of spatial parameters, the optimal number of predators foraging within communication range of each other. Here, we chose to fix *N* and find the optimal group size within that population size. In doing so, we answered the question of whether predators that are already foraging in the same space should do so collaboratively. In these simulation experiments, we chose the total number of predators *N* = 30, and arranged predators into groups of various size, defined using random partitions. Predators within a group were assumed to share all information. Using an agent-level extension of the analytical model (see SOI section “Agent-level Mathematics”), we then computed the expected consumption rate of any one individual predator, as a function of the size of the group it belonged to, over a range of environments defined by the three key timescales *τ*_*h*_, *τ*_*l*_ and *τ*_*s*_. Doing this numerous times, changing the distribution of group sizes at each iteration, allowed us to calculate optimal group sizes for any environment. This produced results qualitatively comparable to other simulation experiments, for example when dividing the entire population into equally sized groups, or when having social ties between all agents and varying *λ* from 0 to 1.

## Results

### Functional Response and the Value of Information

The ABM was first used to explore equilibrium encounter and consumption rates with only two predators in the system, for a range of parameter combinations. The parameter space of the ABM has three essential dimensions—*τ*_*s*_, *τ*_*l*_ and *τ*_*h*_—which can be effectively explored by holding one constant, and systematically exploring the other two. To give a clearer intuitive picture of model results, we present below two choices for the parameters to hold constant, although they can formally be made equivalent. First, we explored prey-handling and first-passage times normalized by a constant landscape mobility, *τ*_*h*_/*τ*_*l*_ and *τ*_*s*_/*τ*_*l*_ respectively. Unsurprisingly, we find that encounter rates (Fig. AA in S1 text) diminish with increasing first-passage times, but they can also show some sensitivity to *τ*_*h*_. Normalized consumption rates *H*/H*—the foraging efficiency—are largest when first-passage times are small, in other words searching for prey is easy, and handling times are high, in other words exploitation is not interrupted by depletion (Fig. AB in S1 text).

Second, we explored various prey handling and landscape change timescales normalized by a constant first-passage time (representing a constant difficulty to find prey), *τ*_*h*_/*τ*_*s*_ and *τ*_*l*_/*τ*_*s*_ respectively (Fig 2). Encounter rates (Fig 2A) are constant unless the landscape timescale *τ*_*l*_ becomes smaller than *τ*_*s*_: this highlights the fact that for high enough prey mobility, encounters do not depend on the predator’s search process, as even a static predator is likely to encounter prey as they move. As for consumption rates (Fig 2B), the symmetry between *τ*_*h*_ and *τ*_*l*_ is made apparent: both timescales limit how long a patch can be exploited, and therefore only the smaller of the two plays a significant role. This dependence of the consumption rate on predator and prey mobility and group behavior—a generalization of functional response accounting for more than prey density—is well reproduced by the analytical model as seen in Fig. C.

**Fig 4:**
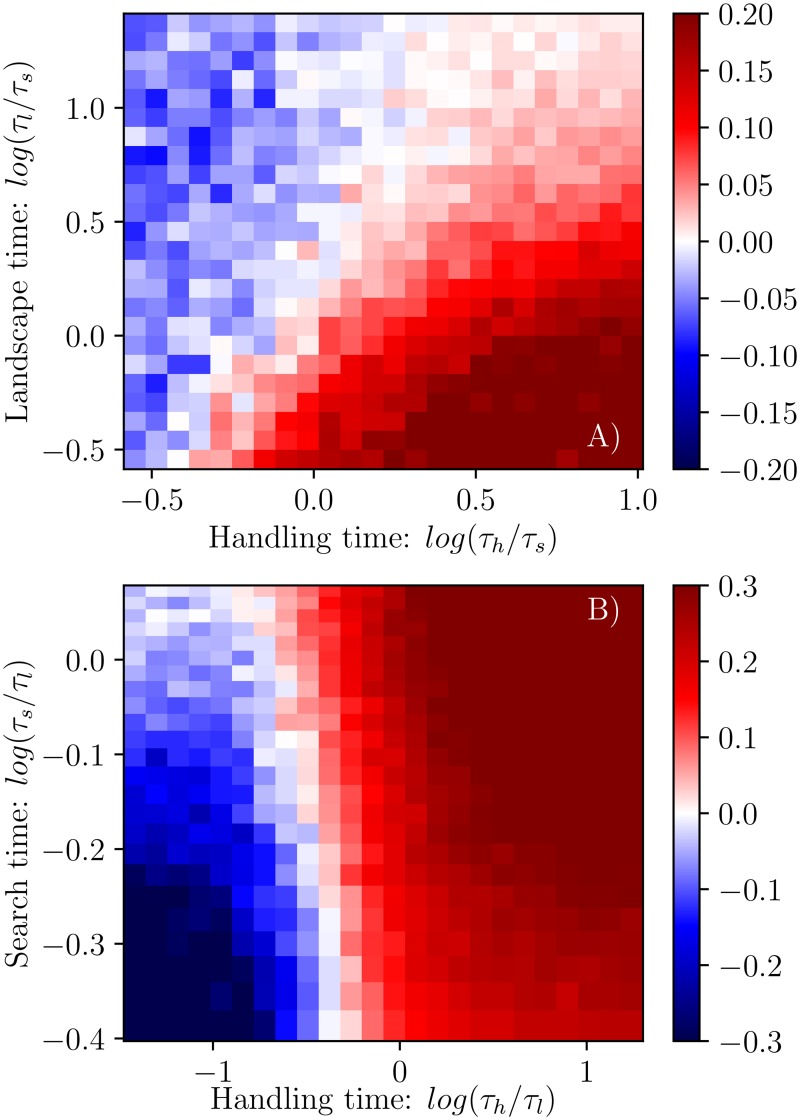
The fractional change of consumption rate between the no-information and full-information sharing cases in a two-predator system. Values are shown for two parameter spaces: the top-panel (A) shows values for a range of prey patch timescales *τ*_*l*_ and handling times *τ*_*h*_, both normalized by the first passage time *τ*_*s*_; the bottom-panel (B) shows values for a range of first passage times *τ*_*s*_ and handling times *τ*_*h*_ normalized by the landscape timescales *τ*_*l*_. Red and blue areas identify parameters combinations (i.e. environments) where information sharing improved and diminished consumption rates respectively. White areas identify environments in which information sharing had no effect.

Throughout the entire parameter space that we explored, information sharing always increased predator encounter rates. The costs of information sharing hinge entirely on having to share prey, which only affects consumption rates. Indeed, information sharing has a highly variable effect on consumption rates, depending on the environment. In the case where the landscape change timescale *τ*_*l*_ is varying with the handling time *τ*_*h*_ (both normalized by the a constant first-passage time *τ*_*s*_:Fig 4A), the value of information is greatest when handling times are long and the landscape change time scale is short. Conversely, the value of information diminishes as handling times decrease and the landscape change time scale increases. Importantly, there is a clear demarcation—a line where the value of information sharing is zero—between environments where it is beneficial to share information and when it is not. In the case where the first-passage time *τ*_*s*_ varies with the handling time *τ*_*h*_ (both normalized by a constant landscape change timescale *τ*_*l*_), we again see the presence of a distinct line of zero value (Fig 4B). Here, there is value to information when handling times and first-passage times are long. and the value of information is least when the handling and first-passage time is short. In both parameter spaces (Fig 4A & 4B) our results are intuitive, and highlight that predators only value information if its benefits are high (search is long) and its cost is discounted by prey mobility: if targets move away before the predators can deplete them together, then there is no penalty to exploiting the same patch.

### Optimal Group Sizes

**Fig 5:**
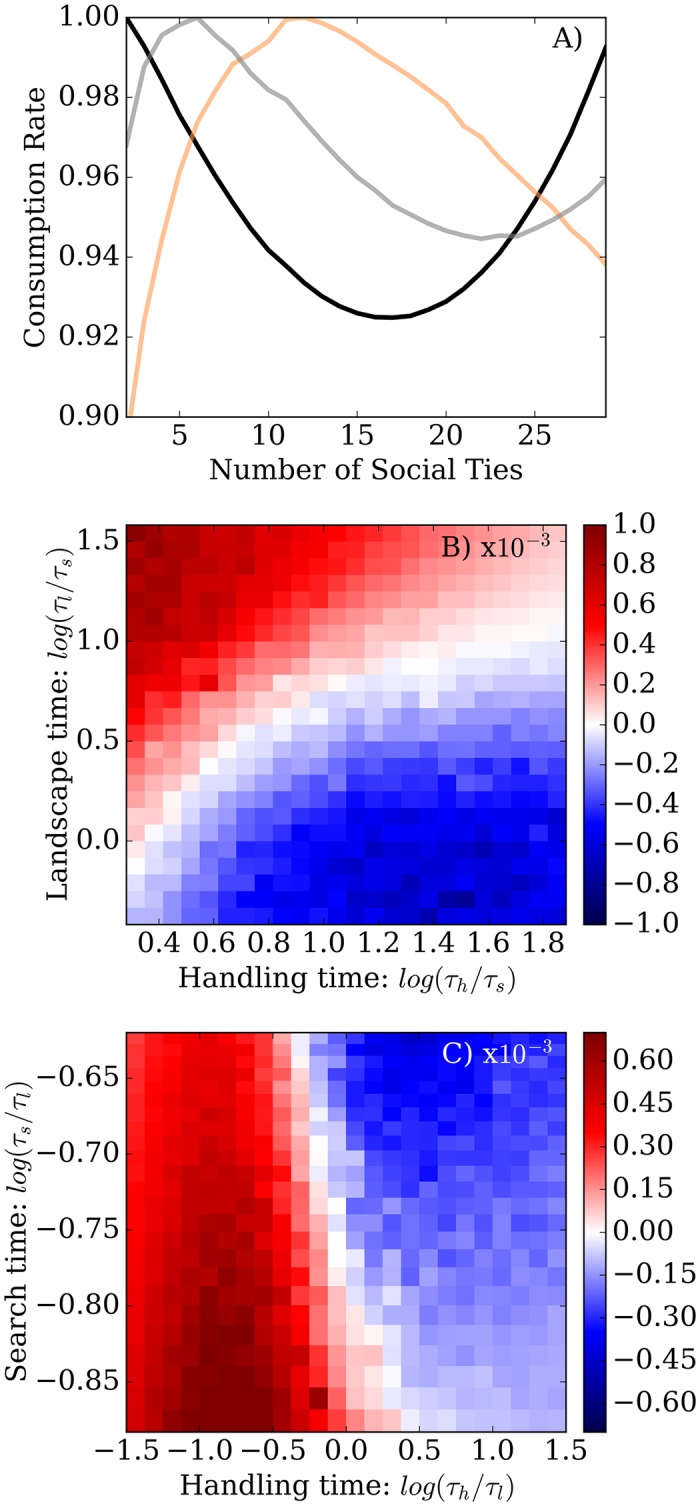
A) Examples of the relationship between expected consumption rate and the number of social ties, for three different environments. These three examples highlight that this relationship can be convex (black line: *log*(*τ*_*h*_/*τ*_*s*_) = 1.08, *log*(*τ*_*l*_/*τ*_*s*_ = 1.5), concave (orange line: *log*(*τ*_*h*_/*τ*_*s*_ = 1.08, *log*(*τ*_*l*_/*τ*_*s*_ = −0.08) and sinuous (grey line: *log*(*τ*_*h*_/*τ*_*l*_ = 0.0, *log*(*τ*_*s*_/*τ*_*l*_ = −0.66). B and C) show the average curvature for this relationship for the normalized landscape timescale *τ*_*l*_ (B) and first passage time *τ*_*s*_ (C) versus handling time *τ*_*h*_ spaces. Red, white and blue areas are those with convex, flat and concave curves respectively.

The optimal group size experiments produced expected consumption rates for individual predators, as a function of group size. Depending on the environment, as defined by the different key timescales, this relationship can be concave, where there is a single optimal number of social ties to have, or convex, where there may be two equally good group sizes to be in. Consider Fig 5A, where three different possible group size curves are shown. In black is an environment in which the curve is convex, and both being alone or operating as one large group are more attractive than anything in-between. As a consequence, despite individual search being the global optimum, fully collective action is also a local optimum. In contrast, the orange curve is concave, revealing an environment in which it is always best to be in a group of intermediate size. Interestingly, other shapes are present, such as the sinuous grey curve, in which there is one global maximum as well as one global minimum, a group size that always performs the poorest.

We calculated the curvature of these relationships (defined as the average of *y*″/(1 + *y*′^2^)^1.5^, where *y* is the consumption rate as a function of group size) over the *τ*_*h*_, *τ*_*l*_ and *τ*_*s*_ spaces (Fig 5B and 5C). In both spaces, there is a clear demarcation between environments in which there is a single optimal group size (negative curvature, blue regions) and those where individual and fully collective action both lead to consumption rates that are far greater than found at intermediate levels of information sharing (positive curvature, red regions). The line separating these regions in the parameter space is close to the one found in the previous figure (Fig 4) between regions of positive and negative value of information in the two-predator simulations. This is striking because it implies that even when information sharing is suboptimal (for example, in the top-left corner of Fig 4A), predators could exhibit high sharing behavior, and not be able to cross over to optimal individual search as intermediate situations are less attractive.

**Fig 6:**
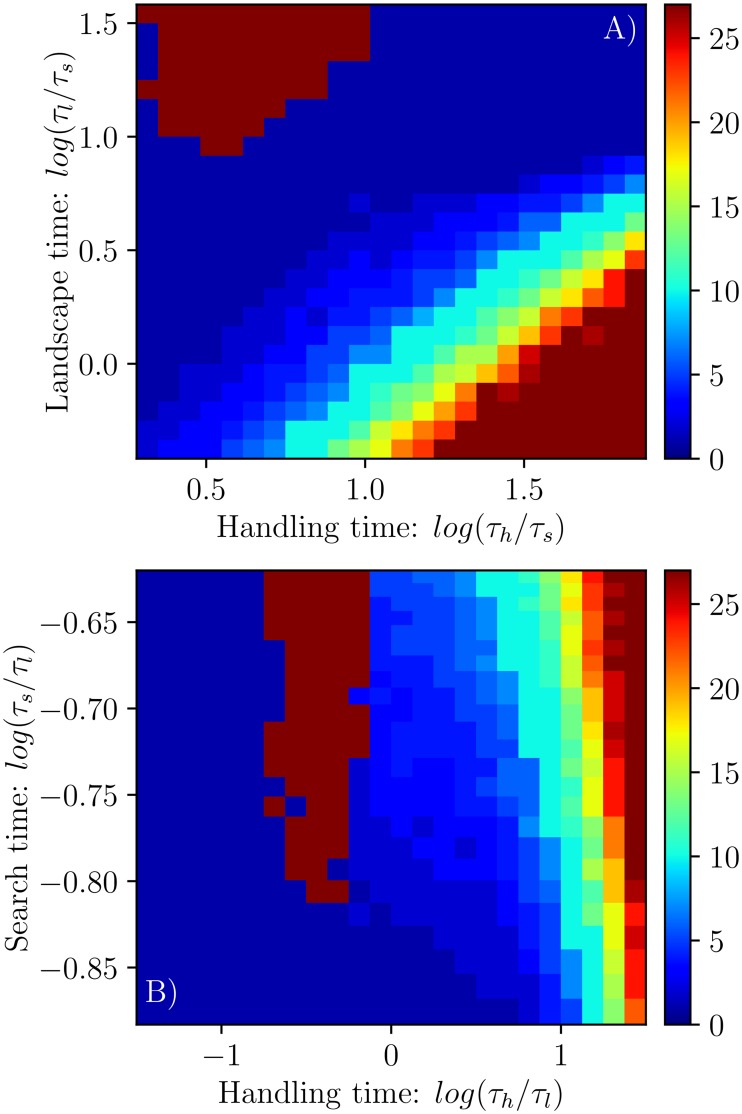
The optimal number of social ties given a total of 30 predators in the system, for (A) the normalized landscape timescale *τ*_*l*_ and (B) first passage time *τ*_*s*_ versus handling time *τ*_*h*_ spaces.

Optimal group sizes (leading to maximum expected consumption rates) were calculated from these curves and are shown for the *τ*_*h*_, *τ*_*l*_ and *τ*_*s*_ spaces in Fig 6A and 6B. For the *τ*_*l*_ and *τ*_*h*_ space, both normalized by a constant *τ*_*s*_, we find that the optimal group size is highest when handling times are high and landscape change timescales are low (Fig 6A). Optimal group sizes then get smaller as handling times decreases and the landscape change timescale increases. However, we find that when handling times are very short and landscape change times are very long, there is possible bistability, as evidenced by the presence of extremely large optimal group sizes (Fig 6A, red blob in the top-left). We observe similar qualitative features in the *τ*_*s*_ and *τ*_*h*_ space, both normalized by a constant *τ*_*l*_ (Fig 6B). In general, optimal group sizes are largest which handling times are large, and are relatively insensitive to changes in the search timescale. The jump to one large super group occurs at intermediate handling times (Fig 6B, the red blob to the left), and at intermediate to large first-passage times. These plateaus of high information sharing occur when the relationship between consumption rate and group size is at its most convex (reflected in Fig 5B & 5C) and it is almost equally good to search either alone or as one large group.

## Discussion

In summary, we have developed a mathematical model of predators searching for and consuming prey, accounting for spatio-temporal heterogeneity and information sharing. The result is a generalized functional response that accounts not only for the density of prey, but also its patchiness and mobility, as well as the number of predators and their behavior in terms of information sharing. We have identified that these factors shape the consumption rate through three key timescales: *τ*_*l*_ the timescale over which the prey landscape changes, *τ*_*s*_ the timescale over which prey patches are found when searching alone, and *τ*_*h*_ the timescale of exploitation of a patch by one predator. These three timescales control the dynamics of a spatially implicit model representing the behavioral states of the predators. The last critical part of this model was *n*_*p*_, the expected number of predators present simultaneously on a patch: it is only through this quantity that spatial and temporal correlations had any significant impact on the output of the model, namely the predators’ consumption rate. Therefore, while the behavioral state abstraction of spatial dynamics is qualitatively robust, for any quantitative agreement to hold, there has to be a satisfactory approximation for *n*_*p*_.

In addition to this mathematical analysis, we developed a computational agent-based model of predators and their prey, accounting for space explicitly and providing a more concrete set of processes. We used both the ABM and the mathematical model to explore a wide swath of parameter space and identified the payoff, either positive or negative, of information sharing for a range of environments. Starting with two predators only, we found that information sharing always improves encounter rates, but reduces consumption rates if prey have low mobility. For a larger number of predators, we found that there is an optimal number (or intensity) of social ties that maximizes consumption rates, going from full collaboration to individual search as prey mobility decreases. However, with low-mobility prey, we found that hunting alone and hunting as one super-group can both be better than intermediate levels of information sharing. Due to the positive curvature of the relationship between individual consumption rates and group-size in that case, selection for optimal group size could exhibit bistability, with full information sharing occurring in regions where individual search was equally or more efficient.

A key step in our analysis was to connect the abstract parameters of the mathematical model to those of the ABM. This allowed us to show that the mathematical predictions made only from the key timescales, matched those of the spatially explicit simulations. We acknowledge that the ABM is still an abstraction of reality, but this choice was necessary in order to compute the key timescales that are central to our analysis. Indeed, we did so using the ABM parameters representing prey density, patchiness and mobility, and predator search patterns, and showed that these estimates were enough to guarantee qualitative and quantitative agreement between the numerical and mathematical results. Within the range of situations covered by our simulation model, we thus found that many of the spatially relevant details had limited impact, as very distinct configurations would lead to identical values for the key timescales and as a consequence predation efficiency. This highlights the complementarity between the two models: the ABM helps give meaning to the few variables entering the mathematical model, while the latter helps predict the consequences of changing simulation parameters or even rules. Certainly, many changes to the ABM would translate to different expressions for the key timescales—for instance, *τ*_*s*_ would be affected by more realistic predator or prey motion. However, these more complex simulations would not change how the key timescales then predict predation efficiency. Hence, the approach developed here is general and can be readily extended to more complex ABMs, better representing specific biological, ecological and social systems.

The key time-scales could also be derived from empirically measured quantities. For instance, the timescale over which a predator consumes a prey patch *τ*_*h*_—the handling time—is a well known quantity in ecology and can be measured for natural and social-ecological systems alike. It is the reciprocal of the rate at which predators consume prey once encountered, which is typically thought of as the time taken to catch, consume and digest prey [33, 34]. Admittedly difficult to measure in the field, there are numerous laboratory estimates of these values for many species [35]. In social-ecological systems, this quantity is possibly easier to measure. For example, for a fishery this value is the expected time it would take for a fishing vessel to either catch an entire fish school or fill its hull [36]. Similarly, the timescale over which prey patches are encountered by a lone predator, the landscape change timescale and the typical distance between predators and their travel speed are all measurable quantities in both natural and social-ecological systems [13, 14].

While the general mathematical model operates at the level of the population, it can be used to derive an agent-level analytical model, allowing us to compare and contrast results directly with the numerical ABM. The derivation of the agent-level mathematics is shown in the SOI section “Agent Level Mathematics”, and these equations provide further intuition about the impacts of information sharing. Indeed, these agent-level equations allow us to address questions of behavioral adaptation or evolution. In exploratory analyses that we performed on predators adapting their social networks, the numerical ABM was far too slow in estimating Evolutionarily Stable Strategies (ESS). In contrast the agent-level mathematics allowed us to compute these ESSs orders of magnitude faster. However, questions of social foraging and evolved cooperation are beyond our scope here, as, in addition to the agent-level mathematics, these analyses require assumptions about how behaviors are selected for [24, 27]. We do not take this next step, and instead simply present our mathematical formulae for the pay-offs of information sharing in different spatial environments.

In all our analyses we have modeled *scramble competition*, the sharing of prey by predators at the same patch, which is just one way in which predators interact [37]. In nature, there are many other possible interactions. For example, two predators consuming prey from the same patch could interfere with one another, diminishing the rate at which prey is consumed [16]. The converse can happen, where per-predator prey consumption rates increase with predator density, for example when it requires multiple predators to catch prey, such as lions and gazelle [2]. So too for humans, for example squid fishers work together by shining lights from their boats into the water [38]. Squid are attracted to the light, and as a consequence, there is a positive relationship between the number of fishers (lights) and consumption rates. These different forms of *within-patch* interactions by predators can be factored into our general mathematical model in the *τ*_*h*_ and *n*_*p*_ terms. For example, if predators interfere with one another as they capture and consume prey, then *τ*_*h*_ will increase. This will have an impact on *n*_*p*_, the expected number of predators consuming prey from a patch, given a certain level of information sharing.

We have also focused on how consumption rates can be maximized through information sharing. But for many systems, the variance in consumption through time is important too. For example, in social-ecological systems, subsistence hunters and fishers are less concerned with maximizing the total amount of money or food they gain. Rather, they are often concerned with avoiding a prolonged state of poverty or hunger [39]. One might assume, then, that information sharing would be a boon to these kinds of predators. However, as we have shown, large levels of information sharing can lead to spatial and temporal correlation in predators (i.e. roaming around as one pack). It is precisely under these social conditions, for certain environments, that predators experience high variance in consumption rate. As a consequence, if minimizing the variance in consumption rates is the objective, then full sharing will not necessarily be selected for. Indeed, human-predators avoid spatial and temporal correlations by, instead of simply sharing information, developing profit- and/or risk-sharing institutions [40] to minimize the variance in consumption.

In our modeling framework we have also assumed that the abundance of prey, either in terms of the number of patches or the number of prey per patch, is stationary and independent of the predators. This reflects systems where the scale at which the predators operate is smaller than that of the prey population. In other words, it is as if our domain is embedded in a larger area describing the dynamics of the prey. However, there are many systems where the scale of the predator population is similar to those of the prey, and as a consequence, predators can have a large impact on coupled demographics [e.g. 41]. This can be accounted for in our general mathematical model, specifically Eq 15. For example, consider a situation where resource depletion diminishes the number of prey patches, while the number of prey per patch remains constant. In such a case, the time between prey patch encounters *τ*_*s*_ will increase. Following Eq 15, this naturally leads to a decrease in consumption rate. Similarly for the situation when the number of patches remains constant, but the number of prey per patch diminishes, then the patch handling time *τ*_*h*_ decreases. Again, following Eq 15, this leads to an increase in the rate at which predators revert to the searching alone state *W*_*L*_, and hence an overall decrease in consumption rate. However, as prey abundance change, so do the incentives to share information. In this case, there are coupled dynamics between the behavior of the predator and the abundance of the prey, with levels of information sharing continually changing as prey abundances change themselves. However, as we have not addressed the (selective) mechanisms behind behavioral change, we do not go any further in studying such a coupled system.

Throughout this paper we have described information sharing in the context of predators and prey in ecological systems, and sometimes in the context of social-ecological systems such as fisheries. However, our results should hold for any system where one actor may benefit from the findings of another. This could be the extraction of natural resources such as oil and minerals by firms, or it could even describe purely social systems, such as dating or finance. The key to linking our results to these other systems, is to identify the analogues to the main dimensions which control the value of information: *τ*_*h*_, *τ*_*l*_ and *τ*_*s*_. Given these quantities, it is possible to hypothesize about the benefits and costs of working together. It is important to note also that all our results could be posed entirely in terms of systems where predators might actively try to conceal their private information. Ultimately, whether information is shared or not, understanding the feedbacks between predator-prey spatial dynamics, and their social preferences, is essential to improving our management of social and ecological systems [8].

## Supporting Information

S1 Text Supplementary information and figures. This document contains all the supplementary information and figures associated with the main text.
(PDF)

S1 Movie Movie showing 1 predator and 10 prey patches that do not move very much (i.e. *τ*_*l*_ is large). (M4V)

S2 Movie Movie showing 1 predator and 10 prey patches that move often (i.e. short *τ*_*l*_). (M4V)

S3 Movie Movie showing 2 predators that do not share information with one another, and 4 prey patches that do not move much. (M4V)

S4 Movie Movie showing 2 predators that share information continuously with one another, and 4 prey patches that do not move much. This movie is paired with M3, and highlights the role that information sharing plays in determining the spatial distribution of predation effort.
(M4V)

S5 Movie Movie showing 2 predators that share information with one another continuously, and 4 prey patches that move frequently. Here the main difference is the sensory radius around the predators (the light red circle around each red predator dot). Increasing the sensory radius reduces the first passage time (i.e. expected time between prey patch encounters) *τ*_*s*_.
(M4V)

S6 Movie Movie showing 10 predators that do not share information with one another, and 4 prey patches that do not move much. Here, the individual prey consumption rate H* is low, hence it takes a long time for one predator to consume an entire patch. But as more predators feed at a given patch, the aggregate feeding rate increases and hence the handling time *τ*_*h*_ diminishes.
(M4V)

S7 Movie Movie showing 10 predators that do share information with one another continuously, and 4 prey patches that do not move much. In such a system the effect of information sharing on the prey patch handling time *τ*_*h*_ is evident. The more predators at a patch, the quicker that patch is consumed.
(M4V)
