# Supplementary material for: The Spatial Dynamics of Predators and the Benefits and Costs of Sharing Information
Source: PLoS Comput Biol. 2016 Oct 20;12(10):e1005147. doi: 10.1371/journal.pcbi.1005147 (PMC5072596; doi:10.1371/journal.pcbi.1005147)
Supplement: S1 Text — This document contains all the supplementary information and figures associated with the main text. (PDF) [file pcbi.1005147.s001.pdf]

Supplementary Information

The Spatial Dynamics of Predators and the Benefits and Costs of Sharing Information

Matthieu Barbier[1#] and James R. Watson[2,3*]

[1]Centre for Biodiversity Theory and Modelling, National Centre for Scientific Research(CNRS), France.

[2]Stockholm Resilience Centre, Stockholm University, Sweden.

[3]College of Earth, Ocean and Atmospheric Sciences, Oregon State University, USA.

Author for correspondence: *james.watson@su.se, #contact@mrcbarbier.org

Population-level Mathematics

At its base, the expected consumption rate of an individual predator can be expressed similarly to the simple behavioral model (Fig. AA, Eq. 2):

$$H = (h + b)H^*, \tag{1}$$

where H^* is the rate at which an individual predator consumes prey items in a patch, and $(h + b)$ is the expected fraction of the predator population feeding (in any patch) in the domain at any given time, and thus also the probability that any given predator is currently consuming prey. The key then to making this calculation is to know the fraction of predators that occupy the behavioral states h and b at steady-state.

While most factors that determine $(h + b)$ are set by the parameters of the prey distribution, two depend crucially on predator behavior: information sharing through λ intuitively reduces the effective search time τ_s, but increases the expected number of predators consuming the same patch n_p, and hence the proportion of a patch that any individual predator might expect to consume. Whichever of these effects dominates in a given environment will decide whether information sharing is good or bad for the predators.

In this mean-field analytical model, we drop all spatially explicit dynamics to get population-wide estimates in the steady state. The spatial distribution of the resource and the details of the search process intervene only inasmuch as they affect the relative value of exploitation and exploration by changing τ_s and n_p. The critical spatial aspect that we need to represent in the model is the emergence of correlation between predators. For instance, when calls are frequent, predators will tend to aggregate into a pack and will spend less time exploring far away from each other. They are thus likely to discover the same prey patches by chance even if there is no call, increasing the expected n_p. To account for this, we introduce one last crucial variable: d

the average distance between predators, or more precisely its rescaled value

$$\tilde{d} = \frac{d}{d_{\max}} = \frac{d}{X\dfrac{\sqrt{2}+\log(1+\sqrt{2})}{6}} \tag{2}$$

where X is the domain diameter and $d_{\max}$ is the expected value of d when predators are completely independent (see our SOI section "Distance between Predators" for a derivation of this equation). Thus $\tilde{d} = 1$ corresponds to case when predators are completely uncorrelated in space, while $d = 0$ corresponds to predators that have aggregated into a single pack. This allows us to define $\tau_d = d/v$, where v is the average speed at which a predator moves.

We finally have the three main variables that specify predator interaction – the expected number of predators consuming prey from a patch n_p, the average distance between predators d and the effective encounter rate $1/\tau_s$, which is the encounter rate accounting for information sharing between predators. We must now express these quantities in terms of model parameters and the behavioral occupation probabilities, to obtain closed-form equations for the latter which we may then solve in the steady state.

Estimating n_p, the expected number of predators at a patch

What follows is an estimation of n_p the expected number of predators consuming the same patch. While a rigorous expression could in principle be derived from a fully stochastic model, an approximation that proves sufficient for quantitative agreement can be derived quite efficiently and intuitively.

We define S_o the total number of occupied patches at any given time. Note that there are on average $N \times h$ predators in the system that have found a patch by themselves. If each of these predators stumbles randomly upon one among S (defined below) available patches, then the number of occupied patches is given by a binomial law that can be approximated by a Poisson process: the number of patches containing at least one predator is

$$S_o \approx S(1 - e^{-Nh/S}). \tag{3}$$

This grows with $N \times h$ and saturates to S the number of available patches. When predators are spatially uncorrelated, $S = N_s$ the total number of patches in the system. But this number should decrease if the distance between predators $\tilde{d}$ is small, as predators are exploring a smaller total area (their search regions overlap) and should encounter each other more frequently. Hence we propose the simplifying assumption that S grows with the explored area, that is proportional to the square of $\tilde{d}$

$$S = N_s \tilde{d}^2. \tag{4}$$

We know that $N(h + b)$ is the expected number of predators that are currently occupying a patch, and we now have S_o the number of occupied patches. Therefore, it is straightforward to define the time-average or unconditional number of predators on a single patch:

$$\tilde{n}_p = \frac{N(h + b)}{S_o}. \tag{5}$$

However, this quantity is distinct from n_p due to spatial and temporal correlation amongst the predators. To illustrate this difference, let us consider the case where all predators share information with one another and ultimately end up moving as one pack. In this case, if a patch is being consumed, it is most likely being consumed by all predators at once and thus $n_p = N$. However $\tilde{n}_p$ contains the likelihood of being on a patch $(h + b)$, which is an average over all time, including when predators are not consuming. Thus

$$n_p \geq \tilde{n}_p \tag{6}$$

and the equality holds only in the uncorrelated limit when the probability of being in either a search or consume phase does not depend on the other agents, i.e. when encounters occur only by random chance. The more correlated the behavioral switches (entering and leaving patches simultaneously), the higher the likelihood that predators will be on the same patch at the same time. Indeed, accounting for these correlations we expect

$$n_p \lessapprox \tilde{n}_p + \lambda(N - \tilde{n}_p) \tag{7}$$

meaning that the number of predators on the same patch is larger than chance, *at most* by $N - \tilde{n}_p$ the number of other predators, times λ, which in this mean-field approach is the probability that each of them was called (we assume that all social ties have equal strength $\lambda \in [0, 1]$). In general the number of predators that can be called is smaller since some of them will be on other patches. A more detailed derivation is given below in the SOI section "Refined Occupancy Approximation".

Distance between Predators

The main text does not consider how behavioral dynamics affect d the typical distance between any two predators, which is useful to quantify one drawback of information sharing: predators explore less of the search space if they form packs. This is a higher-order correction compared to the obvious benefits and costs (finding more patches, but exploiting them faster), nevertheless essential for quantitative agreement with simulation results.

First of all, let us clarify the calculation of the maximal value of d, in the case that predators are totally uncorrelated. In that case, we can assume them to be uniformly distributed over the whole domain. The average distance between any two predators is then

$$d_{\max} = X \int\int_{-1/2}^{1/2} \sqrt{x^2 + y^2} dx dy = X \frac{\sqrt{2} + \log(1 + \sqrt{2})}{6} \tag{8}$$

Now, d may be approximated as follows: let us call ξ the rate at which any predator is called by any other predator

$$\xi = \frac{\lambda}{\tau_s} s^2. \tag{9}$$

We know that the distance to this calling predator, knowing that a call last occurred between them at t_0, can be approximated by a piecewise function:

- $d = \sigma$ if the time since the last call $t - t_0 < 1/W_L$ (i.e. the two agents are in the same patch)
- a diffusive growth $d = \sigma + \sqrt{D(t - t_1)}$ if $t_1 < t < t_2$ with $t_1 = t_0 + 1/W_L$ and $t_2 = (d_{\max} - \sigma)/DD$ such that $d(t_2) = d_{\max}$
- the maximum distance for the fully diffuse state $d = d_{\max} =$ if $t > t_2$, with X the size of the domain

which gives, integrating over all possible values of t_0 given their probability density $e^{-\xi t_0}\xi$,

$$d = \sigma + \sqrt{D\tau_h} e^{-\tau_h \xi} + e^{-\xi\delta/D}(d_{\max} - \sigma - \sqrt{\delta}) \tag{10}$$

$$+ \frac{1}{2}\sqrt{\pi D/\xi}\left(\text{erf}\left(\sqrt{\xi\delta/D}\right) - \text{erf}\left(\sqrt{\tau_h \xi}\right)\right), \tag{11}$$

$$\delta = (d_{\max} - \sigma)^2 + D\tau_h. \tag{12}$$

This expression significantly improves the matching between the analytical model and simulation results, but it makes the former intractable. Therefore, in simpler calculations we need to assume that d is constant, so that we may solve for other quantities first as a function of d, and then solve for d self-consistently in a second time. Eq. (11) is straightforwardly extended to the non-mean-field case which we describe in the SOI section "Agent-level Mathematics", replacing ξ by $\xi_{ij} = s_i c_{ji}$.

Refined Occupancy Approximation

Previously, we presented a simple approximation of n_p the number of predators occupying the same patch. Computing this quantity exactly from a stochastic model is a difficult task. However, we can write a self-consistent equation: we know that $n_p = \tilde{n}_p$ in the absence of correlations, while in the case of full correlation we have $n_p = N$. But n_p itself is a measure of these correlations, which we can use to interpolate between the two values – or more exactly, we can use the fraction of possible neighbors

$$\chi = \frac{n_p - 1}{N - 1} \tag{13}$$

to quantify how correlated behavioral switches are: if $n_p = 1$ predators effectively do not interact, while if $n_p > 1$, either due to chance or calls, there is at least some degree of correlation, because multiple predators

will leave the same patch when it is depleted (or moves away).

We also need to know how many other predators are simultaneously receptive to calls. If predators are uncorrelated, this is N_s the expected number of lone searchers in the steady state; however, in the presence of full correlations, this is at most $N-1$, as they will all become available simultaneously. Thus, if we designate this number by C_a, we can use our measure of correlation χ to interpolate linearly

$$C_a = \chi N + (1-\chi)Ns. \tag{14}$$

Among the predators that have been called, a fraction $1/(1+W_L\tau_d)$ will have reached the patch before it is exhausted or moves away. Then we do a similar interpolation for n_p itself

$$n_p = \chi\lambda\frac{C_a}{1+W_L\tau_d} + (1-\chi)\tilde{n}_p \tag{15}$$

Since χ and W_L depend on n_p (as well as τ_d through d), we get a self-consistent expression for n_p that may be solved numerically, or explicitly if we assume $W_L\tau_d \ll 1$.

Solvable Limits

This section presents various limits in which the model is explicitly solvable. As they represent simplifications of some aspect of the model, they provide insight on which components are necessary to retain the full phenomenology.

We recall that the model can be reduced to an equation on s the fraction of the predator population searching for prey, Eq. 14. Once solved, all other quantities can be recovered using

$$h = s\frac{W_{s\to h}}{W_L}, \tag{16}$$

$$m = s\frac{W_{s\to m}}{W_{m\to b}+W_L}, \tag{17}$$

$$b = m\frac{W_{m\to b}}{W_L}. \tag{18}$$

Fast moving prey $\tau_l \ll \tau_h$

If we can simply assume that $\tau_l \ll \tau_h$ and therefore that exploitation is always interrupted by the preys moving away (rather than the patch being depleted)

$$W_L \approx \frac{1}{\tau_l} \tag{19}$$

then s is trivially given by a root of the quadratic equation Eq. 14 where all the coefficients are now constants – the complexity in the original equations largely comes from n_p in W_L, which is now removed. Likewise if we assume that the number of occupants on a school n_p has little or no variation with $lambda$ and can be taken as a constant.

In that context, information sharing is always good (we can see in Eq. 15 that with W_L constant we have $dH/d\lambda > 0$ everywhere, as only $W_{m\to b}$ and $W_{s\to m}$ depend on λ and both increase with it).

No random interference

Under the condition $N \times h \ll N_s$, we can assume that no two predators find the same patch by chance (it should however be noted that this may break down if predators stay very close together due to frequent calls, i.e. $d \ll d_{\max}$). Hence, the number of occupied patches is simply the number of finders

$$S_o \approx N \times h \tag{20}$$

and the equations above become algebraic (rather than transcendental), provided that we keep d fixed. Furthermore, the uncorrelated number of predators on a patch is $\tilde{n}_p = 1$ and we can use λ itself as a substitute for χ in Eq. (15) since all correlations are created. This approximation may be combined with others below to solve the equation on s explicitly.

However, it should be noted that, in this approximation, one can in principle stack an infinite number of predators in the system if they do not interact socially (i.e. $\lambda = 0$), as they will never encounter each other by chance. Thus, quantities such as the optimal predator population N^{opt}, while explicitly calculable in this

limit, do not take on plausible values if $\lambda \ll 1$.

Fast travel $\tau_d \to 0$

If τ_d is negligible compared to all other timescales, then we can treat the travel time toward the source of a call as negligible, and the m state vanishes. As a consequence

$$\frac{H}{H^*} = h + b = 1 - s \tag{21}$$

and

$$h = \frac{W_{s \to h}}{W_L} s, \qquad b = \frac{W_{s \to m}}{W_L} s, \qquad s = \frac{W_L}{W_L + W_{s \to h} + W_{s \to m}} \tag{22}$$

hence

$$\frac{H}{H^*} = \frac{1}{1 + \dfrac{\tau_s/\tau_l + n_p \tau_s/\tau_h}{1 + \lambda N (1 - H/H^*)}} \tag{23}$$

which looks like a natural generalization of Eq. 2 with the added effects of landscape mobility and communication.

This is still not solvable analytically unless we also assume the absence of random interference (see above), in which case the system becomes algebraic and of low degree given a sufficiently simple approximation for n_p. Thus, explicit solutions can be computed; but they are very cumbersome and bring little new information: given the two strong assumptions they require, they do a poor job at recovering features of the numerical results quantitatively.

However, even assuming that there is random interference, it is easy to see under which conditions information sharing is beneficial or not. Given the equation above, it appears that communication is counter-productive if it increases the probability of being in the searching state, i.e.

$$\frac{d}{d\lambda} \frac{1 + \lambda N s}{\tau_s/\tau_l + n_p \tau_s/\tau_h} < 0, \tag{24}$$

meaning that $1 + n_p\tau_l/\tau_h^1$ should exhibit faster relative increase with λ than $1 + \lambda N_s$. Since n_p increases at least as fast as λNs, a simple condition to ensure this is the obvious $\tau_l/\tau_h^1 > 1$. Whether n_p is concave or convex can decide whether the consumption rate has a minimum or a maximum for $0 < \lambda < 1$, as is intuitive once again: a minimum is likely to occur if n_p increases faster at low λ, meaning that predators starting to share information encounter the costs faster than the benefits even if a higher level of sharing is eventually beneficial. The basic approximation given for n_p in the main text is linear in λ and thus lacks the potential for such effects.

Agent-level Mathematics

The population-level mean-field model described above assumes that all agents are connected in a complete graph of social ties with equal strength. This allows us to examine how overall levels of information sharing change the average consumption rate over the predator population. However, if we are to gain a deeper understanding of how social networks change in response to environmental dynamics, we must move back to the level of the individual and allow for heterogeneity, such as different numbers of ties and tie strengths. Adapting the mean-field model to an arbitrary social network is rather straightforward: the steady-state consumption rate and probabilities for the four behavioral states, which were previously equal for all predators, must now be computed for each predator individually, but the structure of the equations remains very similar.

Starting with each predator, indexed by i, we thus assign individual probabilities of being in any behavioral state: s_i, h_i, m_i and b_i, and define λ_{ij}, which is the edge from predator i to j, describing how likely the former is to transmit information to the latter – once again, it can be binary or weighted in the range $[0, 1]$. While the expressions below account for the fact that these ties may be asymmetric, reflecting e.g. spying and other non-reciprocal aspects of information sharing, in all later simulations we will impose $\lambda_{ij} = \lambda_{ji}$ even as those ties evolve. The rates at which individual predators switch behavioral states can be computed using variants of the mean-field equations where every occurrence of N must now be replaced by a sum over neighbors:

$$W^i_{s\to h} = \frac{1}{\tau_s} \tag{25}$$

$$W^i_L = \frac{1}{\tau_l} + \frac{n_{o,i}}{\tau_h} \tag{26}$$

$$W^i_{s\to m} = \sum_j c_{ji} = \sum_j W^j_{s\to h}\lambda_{ji}s_j \tag{27}$$

$$W^i_{m\to b} = \frac{d_i}{v}, \tag{28}$$

where we introduce c_{ij} the rate at which i shares information with j. It is used in computing d_i, the expected distance between predator i and its neighbors in the social network, i.e. other predators sharing information with it:

$$d_i = \frac{\sum_j c_{ji}d_{ij}}{\sum_j c_{ji}} \tag{29}$$

and d_{ij} is the expected distance between predators i and j. The calculation of d_{ij} follows that of d in the mean-field case as described above the SOI section "Distance between Predators": $n_{o,i}$ the number of predators consuming jointly with predator i follows an equation similar to (15), but now including o_{ij} the probability that predator i and j consume prey from the same patch at the same time:

$$n_{o,i} = 1 + \sum_{j\neq i} o_{ij} \tag{30}$$

$$o_{ij} = \lambda_{ij}\left(1 - (1 - o_{ij})(1 - s_j)\right) + (1 - o_{ij})\,\frac{h_j + b_j}{S_o} \tag{31}$$

$$o_{ij} = \frac{\lambda^2_{ij}s_j + \dfrac{h_j + b_j}{S_o}}{1 - \lambda^2_{ij}(1 - s_j) + \dfrac{h_j + b_j}{S_o}} \tag{32}$$

where the number of occupied patches is

$$S_o = N_s(1 - e^{-\sum_i h_i/N_s}). \tag{33}$$

We then search numerically for vectors $\vec{s}$ and $\vec{n}_p$ that satisfy the following conditions: all behavioral state change probabilities must sum to unity:

$$1 = s_i + h_i + m_i + b_i, \tag{34}$$

while $n_{o,i}$ the number of predators consuming the same patch as predator i is also computed by analogy with the mean-field expression, and can be approximated by

$$n_{o,i} \approx \sum_j \frac{h_j + b_j}{S_o} + \sum_j \left(\frac{\lambda_{ij} + \lambda_{ji}}{2} - \frac{\lambda_{ji} h_j + \lambda_{ij} b_j}{S_o} \right) \tag{35}$$

where the first term corresponds to random encounters on the patch, and the second considers, for each other predator j, the possibility that it is either in the free consuming state and called by predator i, or was called by i and is now in the bound consuming state.

In the SOI section "Validation of Analytical Results" we show that the expressions from both the mean-field and agent-level mathematical analysis create results that quantitatively match the results produced from the full numerical implementation of the ABM. This validates their use in exploring systems with large numbers of predators.

Validation of Analytical Results

The mathematical analysis was developed to complement the numerical simulations of the ABM in two ways. First, we needed to reduce the vast parameter space presented in Table 2 to a few variables that may be explored systematically. Using a nondimensionalization approach [1], we propose that prey and predator

parameters enter the expression of the average consumption rate through only four timescales: τ_s for search, τ_h for consumption, τ_l for landscape mobility and τ_d for travel between predators. Together, these four processes encompass all the dynamics of the system, and we assume that they can all be understood as Poisson processes and parametrized by their typical timescale. For any given social network, the expected consumption rate (nondimensionalized using its maximal value H^* which corresponds to consumption while on a patch), can thus be written as

$$\frac{H}{H^*} = \mathcal{H}(\tau_s, \tau_h, \tau_l, \tau_d | \text{Social network}). \tag{36}$$

Of these four timescales, the first three are clearly necessary to understand the costs and benefits of information sharing in a changing environment. On its own, τ_d may seem less important: a long travel time to the source of a call reduces the benefit of using information, but the limit $\tau_d \to 0$ can in principle be taken without affecting the dynamics dramatically. However, removing it entirely causes a notable degradation of quantitative agreement between analytical and simulation results; most of the time, we will consider it to be non-zero but slowly varying as a function of the others. This leaves only three timescales, from which two nondimensional ratios can be constructed. Given a social network, or its two defining parameters N and λ in the mean-field case, these two timescale ratios are the only dimensions that we need to explore. If one such ratio is fixed, then all quantities in the system, once properly nondimensionalized, should depend at most on the other ratio, as we indeed confirm from simulations, see Fig. D.

We also note that τ_l acts mainly as a cutoff on the other timescales: if $\tau_h/N \gg \tau_l$, prey patches move away before they can entirely be consumed even by all predators together, and the consumption/handling time is thus always τ_l; if $\tau_s \gg \tau_l$, the search process becomes irrelevant as the landscape moves faster than it is being searched, and encounters thus occur by chance on a timescale τ_l/ϕ given the fraction $\phi \approx N_s \pi \sigma^2 / X^2$ of the domain covered by prey patches.

We then developed an analytical model to reproduce the phenomenology of the ABM, with sufficient accuracy that it may replace computationally expensive simulation runs when we wished to investigate new directions such as the evolution of the social network. While this theoretical model lacks most of the fine-grained details of the ABM, and in particular contains no explicit spatial dimension, its predictions are mostly in

good quantitative agreement with ABM simulation results. They fall within $\sim 10\%$ of each other in most regions of the explored parameter space, see Fig. C.

Intermittent Search

In order to derive the search time τ_s from predator and prey characteristics, we take advantage of Benichou et al. 2011 [2], who analyzed intermittent search in a simpler setting: a single "predator" searching a circular domain for a single target. We recall a result from that work for the *first-passage time*, or expected time before encountering the target:

$$\tau_s = \frac{1+\tau_b}{2z^2}\left(\frac{\xi}{a}(z^2-a^2)^2\frac{I_0(a\xi)}{I_1(a\xi)} + \frac{\xi^2}{4}\left(4z^4\log(z/a)+(z^2-a^2)(a^2-3z^2+8/\xi^2)\right)\right), \tag{37}$$

where τ_b is the expected time spent moving ballistically, a is the detection radius of the predator, z is the radius of the search domain, $I_0(\cdot)$ and $I_1(\cdot)$ are modified Bessel functions of the first kind and $\xi = \sqrt{2}/(v\tau_b)$. In our system, $\tau_b = 1/r_p$ (with r_p the turning rate), $a = \sigma + \sigma_s$ the sum of the predator sensing radius and the prey patch radius, and we propose the following extension for multiple prey patches

$$z = a + \frac{X}{2\sqrt{N_s}}, \tag{38}$$

with X the diameter of the whole domain, and N_s the number of patches in the system. By factoring the number of prey patches N_s in such a way, we simply model the effective distance that a predator has to search in order to find a patch as decreasing with the number of patches in the system.

As exposed in the main text, we assume that predators search optimally given a prey distribution (N_s and

σ): more specifically, we fix v and σ_s beforehand, then select the turning rate r_p that maximizes the rate at which prey patches are encountered. However, for the sake of analytical tractability, we use the single predator optimum. Given the formulation above for the first passage time, the turning rate r_p that minimizes it is given by:

$$\frac{1}{r_p} = \frac{a}{v}\sqrt{\log\left(\frac{z}{a}\right) + \frac{1}{2}}. \tag{39}$$

These equations follow the analysis presented in Benichou et al. 2011 [2] and allow us to calculate optimal turning rates for predators (searching alone) in any environment (as defined by the parameters of the model).

The analytical formula for the optimal turning rate was tested against numerical simulations of the ABM, parameterized for one predator, and the results agree (Fig. EA), confirming its use in determining the optimal turning rate as well as the expected prey patch encounter rate. The slight discrepancy can probably be ascribed to a local depletion effect described in the SOI section "Local Depletion Effect". These initial 1-predator simulations also explored how changing the sensory radius of the predator σ_s affects the search time (Fig. EB), as this is the main control variable that we have been using to explore the range of search times.

Local Depletion Effect

One important source of discrepancies is a *local depletion effect* that appears in the ABM and is not taken into account in the analytical model. If predators diffuse slowly enough compared to the handling time τ_h, they will spend a significant time searching and consuming in a limited region. Although preys are in principle distributed uniformly, that region will become locally depleted due to a stochastic effect: prey patches will more frequently jump away (due to being exhausted) than jump in the region from other places, which means that the region will have a negative net outflux of prey. In that limit, we thus expect lower catch rates than predicted by the analytics, and furthermore, that predators will do better if they move further away to escape the depleted region, which translates into a lower-than-predicted optimal value for r_p the turning rate in

intermittent search. Those expectations are both confirmed in Fig. F, with excellent agreement for optimal r_p when the simulation is interrupted before the local depletion effect can become significant (as it requires predators to stay in the same region for long enough).

In turn, the magnitude of the effect allows to quantify the size of the region being steadily depleted by a predator. Let us assume that it takes place over a region of area σ surrounding the predator, containing prey density ρ out of a domain of area 1 with average density 1. Let us denote by $\bar{\sigma}$ the area excluding σ, whose density $\bar{\rho}$ is given by

$$\bar{\rho}(1-\sigma)+\rho\sigma = 1. \tag{40}$$

Assuming $\tau_h < \tau_l$, the fluxes leaving and entering that region are given by the rates of school movement or depletion times the probability of landing in each region after a jump, i.e.

$$W_{\sigma\to\bar{\sigma}} = \frac{1-\sigma}{\tau_h} \tag{41}$$

$$W_{\bar{\sigma}\to\sigma} = \frac{\sigma}{\tau_l}. \tag{42}$$

The equilibrium density of prey surrounding the predator ρ is given by the fact that these fluxes multiplied by the corresponding densities must be equal,

$$\rho W_{\sigma\to\bar{\sigma}} = \frac{1-\rho\sigma}{1-\sigma} W_{\bar{\sigma}\to\sigma} \tag{43}$$

hence

$$\rho = \frac{1}{\sigma + (1-\sigma)\dfrac{W_{\sigma\to\bar{\sigma}}}{W_{\bar{\sigma}\to\sigma}}} \tag{44}$$

$$= \frac{\sigma}{\sigma^2 + (1-\sigma)^2\dfrac{\tau_h}{\tau_l}}. \tag{45}$$

Finally, since ρ is equal to the ratio of H under the depletion effect to H without, we can measure as the

ratio of the lowest to the highest curve on Fig. F and invert the equation above to estimate

$$\sigma = 1 + \frac{1 - 2\rho - \sqrt{1 + 4(1-\rho)\rho\tau_h/\tau_l}}{2\rho(1+\tau_h/\tau_l)}. \tag{46}$$

We find that the radius corresponding to this area is $r \approx 1.3(\sigma + \sigma_s)$, with a slow dependence in r_p for the prefactor. This falls within the range of intuitive expectations, as $\sigma + \sigma_s$ is the effective detection range of the predator (to the center of a prey patch).

References

[1] Barenblatt GI. Scaling, self-similarity, and intermediate asymptotics: dimensional analysis and intermediate asymptotics. vol. 14. Cambridge University Press; 1996.

[2] Bénichou O, Loverdo C, Moreau M, Voituriez R. Intermittent search strategies. Reviews of Modern Physics. 2011;83(1):81.

Supplementary Movies

We refer the reader to a set of movies showing (qualitatively) the dynamics of the fully numerical agent-based model. These movies show:

M1) movie showing 1 predator and 10 prey patches that do not move very much (i.e. τ_l is large)

M2) movie showing 1 predator and 10 prey patches that move often (i.e. short τ_l)

M3) movie showing 2 predators that do not share information with one another, and 4 prey patches that do not move much.

M4) movie showing 2 predators that share information continuously with one another, and 4 prey patches that do not move much. This movie is paired with M3, and highlights the role that information sharing plays in determining the spatial distribution of predation effort.

M5) movie showing 2 predators that share information with one another continuously, and 4 prey patches that move frequently. Here the main difference is the sensory radius around the predators (the light red circle around each red predator dot). Increasing the sensory radius reduces the first passage time (i.e. expected time between prey patch encounters) τ_s.

M6) movie showing 10 predators that do not share information with one another, and 4 prey patches that do not move much. Here, the individual prey consumption rate H^* is low, hence it takes a long time for one predator to consume an entire patch. But as more predators feed at a given patch, the aggregate feeding rate increases and hence the handling time τ_h diminishes.

M7) movie showing 10 predators that do share information with one another continuously, and 4 prey patches that do not move much. In such a system the effect of information sharing on the prey patch handling time τ_h is evident. The more predators at a patch, the quicker that patch is consumed.

Supplementary Figures

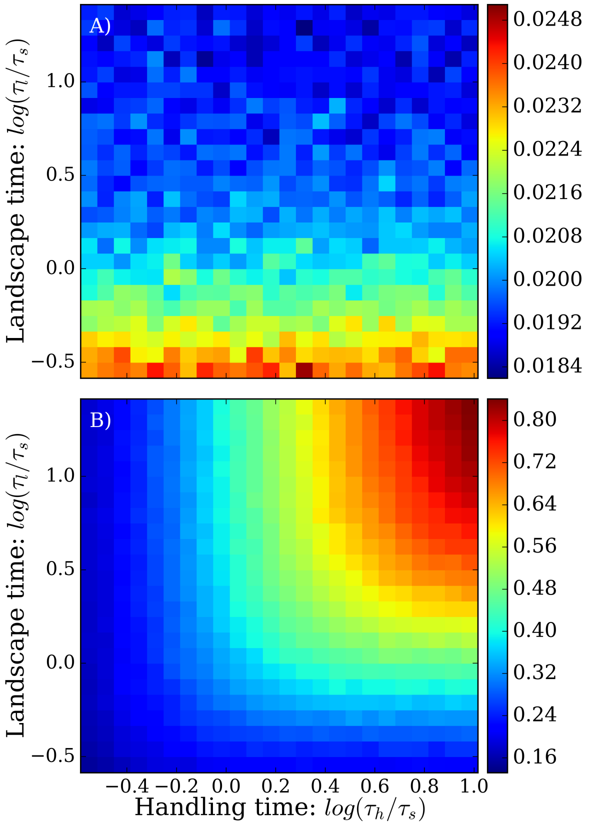

Figure A. Expected encounter (A) and normalized consumption (B) rates for a system with two predators. Here, values are shown when varying the times that prey patches stay in one place τ_l, and the prey patch handling time τ_h, both normalized by the expected time between prey patch encounters for one predator τ_s (which is constant across realizations).

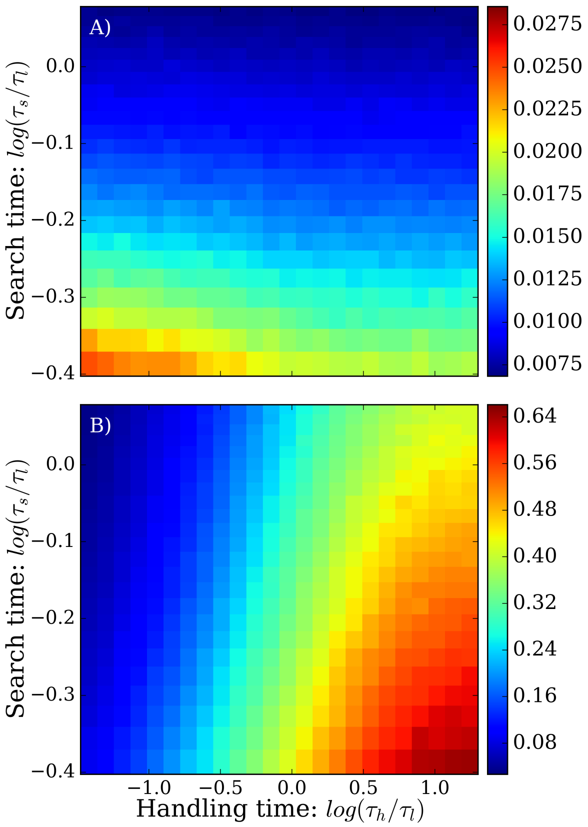

Figure B. Expected encounter (A) and normalized consumption (B) rates for a system with two predators. Here, values are shown when varying the expected time between prey patch encounters for one predator τ_s, and the prey patch handling time τ_h, both normalized by the time that prey patches stay in one place τ_l (which is constant across realizations).

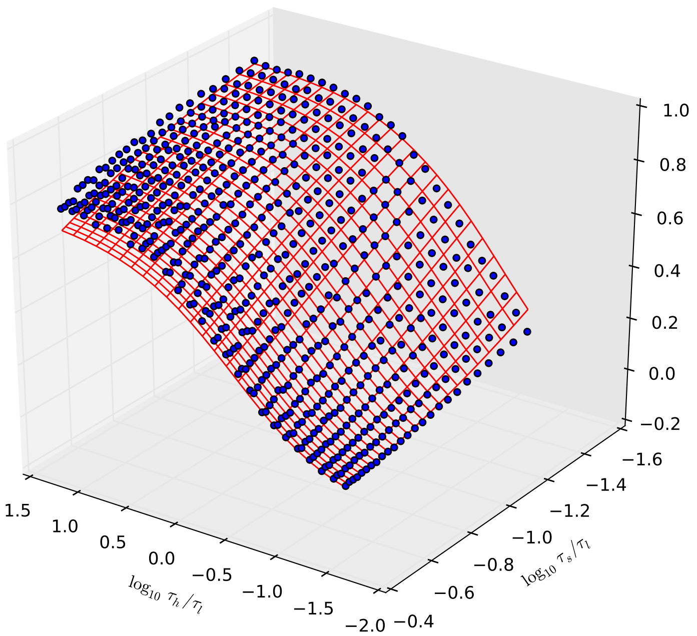

Figure C. Feeding functional response and analytical model validation: the vertical axis is the foraging efficiency H/H^* calculated from the analytical model (wireframe) and from the ABM simulation runs (dots) as a function of the key timescale ratios (horizontal axes). These values remain within 10-20% of each other, suggesting our simplified mathematical analysis captures the main features of the ABM.

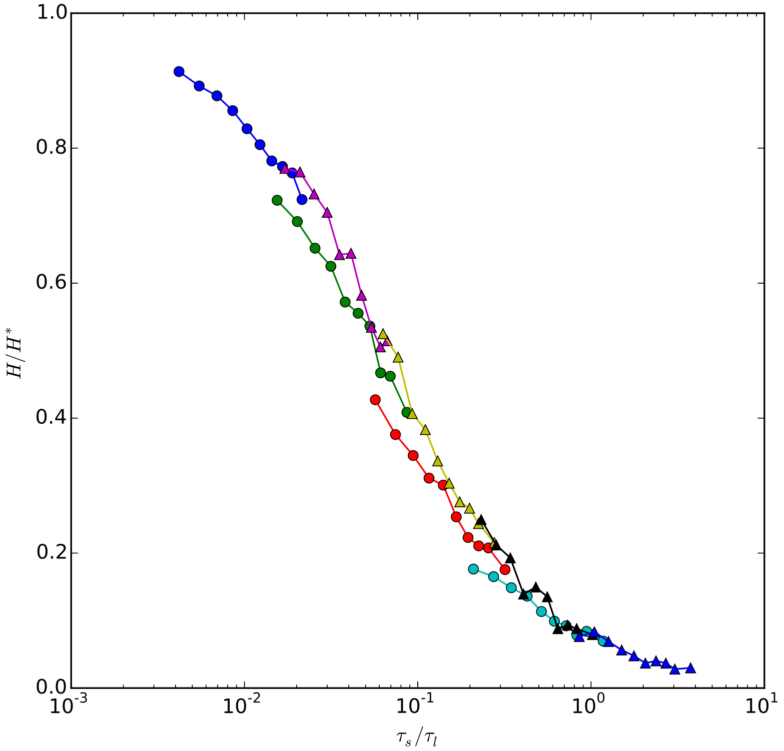

Figure D. Validation of the nondimensionalization approach. Each curve gives the rescaled consumption rate H/H^* observed in the ABM simulation runs, for various samplings of the vast parameter space, where the ratio τ_l/τ_h is maintained. In the SOI section "Validation of analytical results", we clarify that in this case, any nondimensional quantity of interest, including H/H^*, should then depend only on the ratio τ_s/τ_l (provided that travel between predators is fast, i.e. τ_d is negligible). Indeed, we see that all the various curves collapse on a single curve depending on that ratio, with small discrepancies that may be explained by a slow dependence in τ_d. Here, we show two settings: circles correspond to $N_s = 18$ and $\sigma = 3$ (many small prey-patches), triangles correspond to $N_s = 6$ and $\sigma = 9$ (few large prey-patches). For each setting, four curves are plotted, corresponding (from left to right) to $r_l = [0.001, 0.004, 0.014, 0.05]$. Each curve is obtained by letting σ_s vary from 2 to 9, and adapting H^* so that, in every simulation, the prey have the same effective mobility $\tau_l/\tau_h = 10$

.

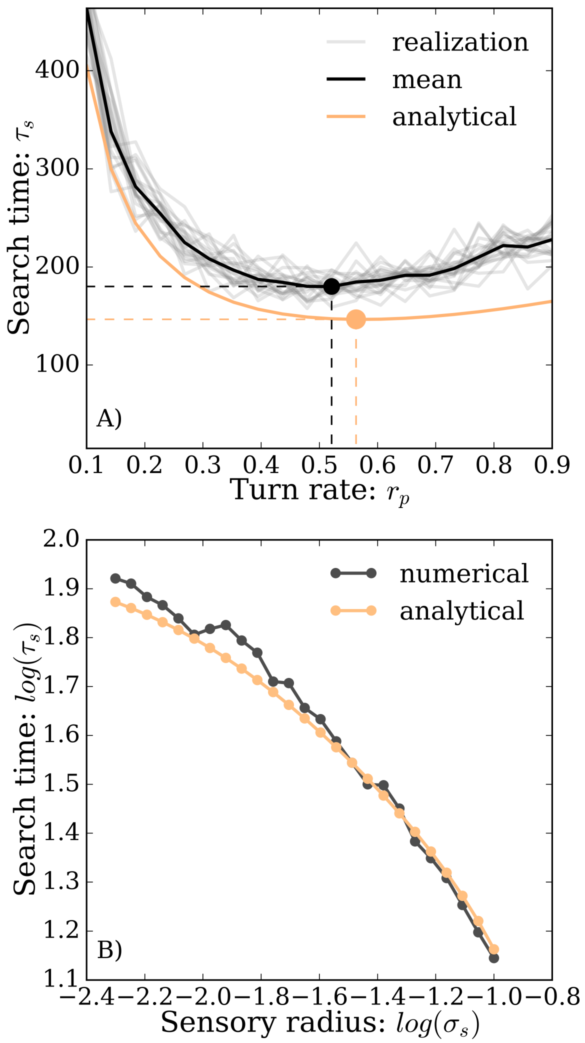

Figure E. A) The time between prey patch encounters changes as the probability per unit time of changing direction (and locally sensing the environment) r_p is varied. The optimal turning rate $(1/r_p)$ is the one that minimizes this time (and hence maximizes the encounter rate). B) The time between prey patch encounters (given one predator in the system: τ_s) changes as the predator sensory radius is increased. In both the top and bottom panels, the numerical results (black lines) are matched by our analytical approximation (orange lines).

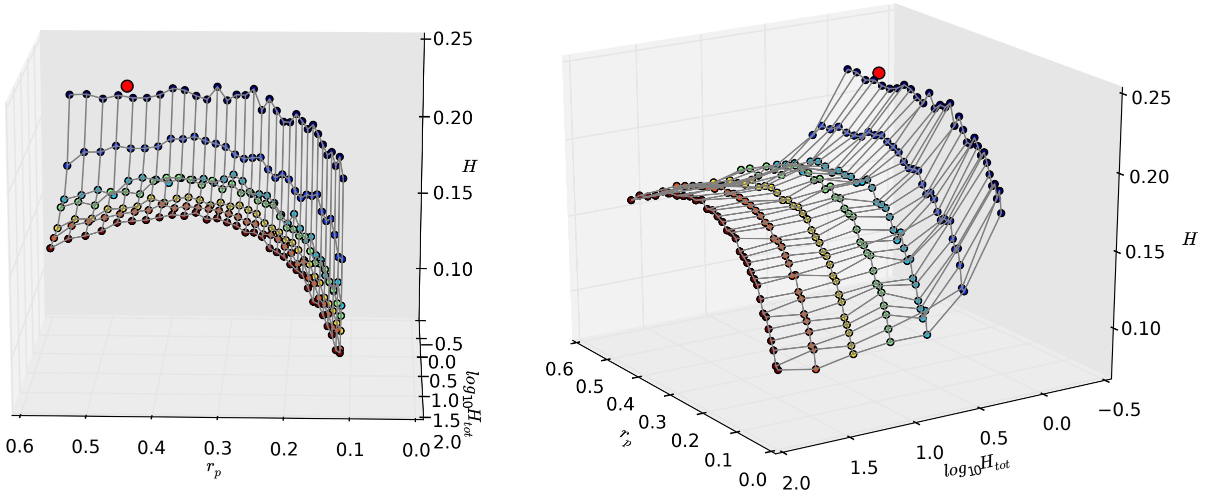

Figure F. A study of the local depletion effect: two snapshots of consumption efficiency H/H^* produced from the ABM simulations, as a function of r_p the turning rate in abscissas and H_{tot} – the total amount caught before the simulation is interrupted. It is evident that consumption efficiency is maximal early in the simulation, then converges to a lower value. The optimal value of r_p at long times (around 0.35) is smaller than at short times. The analytical prediction for the optimal turning rate (red dot) is $r_p = 0.46$, and thus is in agreement with the short time result. Note - the color of the markers has no quantitative meaning, and is simply used for visual clarity.
